# Decoding of Superimposed Traces Produced by Direct Sequencing of Heterozygous Indels

**DOI:** 10.1371/journal.pcbi.1000113

**Published:** 2008-07-25

**Authors:** Dmitry A. Dmitriev, Roman A. Rakitov

**Affiliations:** Illinois Natural History Survey, Champaign, Illinois, United States of America; Washington University, United States of America

## Abstract

Direct Sanger sequencing of a diploid template containing a heterozygous insertion or deletion results in a difficult-to-interpret mixed trace formed by two allelic traces superimposed onto each other. Existing computational methods for deconvolution of such traces require knowledge of a reference sequence or the availability of both direct and reverse mixed sequences of the same template. We describe a simple yet accurate method, which uses dynamic programming optimization to predict superimposed allelic sequences solely from a string of letters representing peaks within an individual mixed trace. We used the method to decode 104 human traces (mean length 294 bp) containing heterozygous indels 5 to 30 bp with a mean of 99.1% bases per allelic sequence reconstructed correctly and unambiguously. Simulations with artificial sequences have demonstrated that the method yields accurate reconstructions when (1) the allelic sequences forming the mixed trace are sufficiently similar, (2) the analyzed fragment is significantly longer than the indel, and (3) multiple indels, if present, are well-spaced. Because these conditions occur in most encountered DNA sequences, the method is widely applicable. It is available as a free Web application Indelligent at http://ctap.inhs.uiuc.edu/dmitriev/indel.asp.

## Author Summary

In DNA, information is encoded as a sequence of four types of building blocks–nucleotides. The most common technique for determining such sequences, the Sanger method, outputs a single consensus for a pool of DNA molecules in the analyzed sample. When these are identical, each site in the output contains a single nucleotide call. Yet, samples from organisms with two sets of chromosomes generally contain two types of DNA molecules (alleles), each derived from one parent. If, due to insertion or deletion (indel) mutations, one allele contains extra nucleotides, most sites in the sequencing output beyond the mutation site will contain pairs of nucleotide calls. While signaling the presence of a potentially important mutation, such output cannot be read directly and often gets discarded. Here we describe an algorithmic method which accurately reconstructs the pair of allelic sequences from the observed complex pattern of calls. Unlike most existing computational approaches to the problem, our method does not require knowledge of one of the involved sequences to use as a reference, nor any other additional information. Therefore, it can facilitate sequencing of indel-rich regions of genomes and speed up discovery and characterization of indel mutations, including those causing diseases in humans.

## Introduction

**Figure 1:**
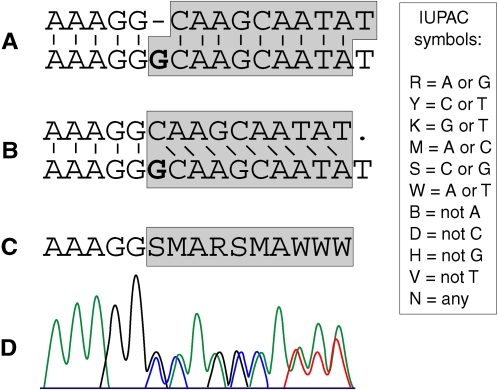
A pair of allelic sequences properly aligned (A), unaligned (B), and translated into a consensus (C). The trace resulted from direct sequencing of the pair is shown in (D). The one-base insertion is shown in bold face. Links between the allelic strings represent positional homologies. The bases forming mixed trace are highlighted with grey. The standard IUPAC symbols for 2-fold degenerate DNA bases are enclosed in the box.

Direct fluorescent sequencing of two dissimilar templates produces a mixed trace, which appears as if the traces obtained for each template separately were superimposed onto each other. Simultaneous sequencing of completely unrelated templates occurs during sequencing of RT-PCR products containing alternative splicing sites and during screening of random insertional mutagenesis libraries [1]. More often mixed traces occur as a result of direct sequencing of diploid alleles containing heterozygous insertions/deletions. In this case, the mixed trace downstream of the indel is formed by two allelic traces superimposed onto each other with a phase shift [1]–[5] (Figure 1). Mixed traces are often discarded as uninterpretable. New sequencing technologies, such as pyrosequencing, avoid the problem by working from single DNA molecules [6], but these emerging methods still have limited application [7]. In traditional capillary electrophoresis sequencing, the problem can be avoided by separating the templates prior to sequencing via cloning into a vector or by selectively amplifying one allele using allele-specific primers. Because these solutions are costly, several computational methods have been developed to extract information from mixed traces.

**Figure 2:**
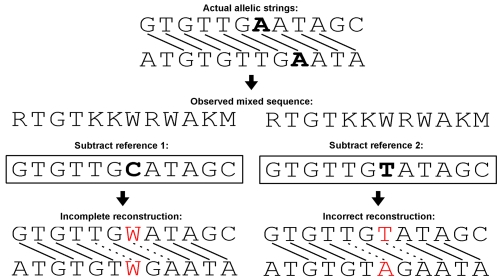
Examples of situations when the reference-based approach results in incomplete or incorrect reconstructions of a mixed sequence. Links between bases of the actual allelic strings (top) indicate positional homologies. The chosen reference sequences each differ from the top allelic string at one site (bold letters). Subtraction of Reference 1 results in one site in each reconstructed allelic string remaining unknown (red letters). Subtraction of Reference 2 results in one incorrectly reconstructed site in each allelic string (red letters). Note that, in the last case, the reconstructed fragment is heterozygous at two sites (dashed homology links). For the meanings of the IUPAC symbols see Figure 1.

Most of these methods require knowledge of a reference sequence, *i.e.*, a sequence believed to be identical to one of the two mixed templates [2]–[4]. Algorithms for “subtracting” from the mixed sequence a reference sequence, supplied by the user, have been incorporated into several software packages, including PolyPhred [4], STADEN package [8], CodonCode Aligner (CodonCode Corp., Dedham, MA, USA), Mutation Surveyor (SoftGenetics), novoSNP [9], InSNP [10], PolyScan [11], and AutoCSA [12]. This approach has been used to detect and characterize sequence variants in clinical applications, such as detecting somatic heterozygous variants in primary cancers [12], and to discover rare indel polymorphisms in large-scale resequencing projects [3]. A similar algorithm has been recently developed, which uses as a reference the best matching genomic sequence obtained by aligning the mixed sequence to the appropriate genomic database [1]. The reference-based methods decode mixed traces formed by related (allelic) as well as completely unrelated templates, but the requirement of a reference restricts their use mostly to extensively sequenced organisms and loci. Moreover, the assumption that the chosen reference sequence should be identical to one of the unknown templates comprising the mixed trace may not always hold true, potentially leading to errors in reconstruction (Figure 2).

A different approach is used by the proprietary algorithm in SeqScape and Variant Reporter (both Applied Biosystems Inc., Foster City, CA, USA), which detects and decodes single heterozygous indels without a reference sequence, but only when mixed traces produced by both direct and reverse sequencing of the same template are available ([13] and a personal communication of an AB employee). Recently, Flot et al. [5] developed an elegant method for deconvolution of mixed traces, which also uses the direct and reverse sequences of the same template. Implemented as the web software Champuru [14], the method is based on the observation that, as long as two templates differ in length, the direct and reverse sequences of their mixture provide complementary information, which can be combined to fully restore the original template sequences even if these were unrelated.

To our knowledge, the only tool developed so far to extract superimposed sequences from an individual mixed trace is the web application ShiftDetector [15] (While this manuscript was in production, the authors became aware that a proprietary algorithm capable of decoding individual mixed traces resulted from single indels up to 25 bp had been recently included in CodonCode Aligner Version 2.0 by CodonCode Corp., Dedham, MA, USA). To detect heterozygous indels, the program processes a trace file and estimates for each site the probability that peaks at the next 10 sites have resulted from a phase shift between 0 and 25 bp by recording how many of these peaks are repeated at the corresponding distance downstream in the trace. Instead of a pair of allelic sequences, the program reconstructs a single consensus sequence, beginning at variable distances downstream of the indel, which itself remains undecoded. Moreover, in this study we found that under ideal conditions ShiftDetector decodes only 56.0 to 85.5% of the ambiguous sites present in the input trace. Apparently due to these shortcomings, the method has found only limited use [16].

Similarly to ShiftDetector, the method we describe here decodes superimposed allelic sequences solely from the complex pattern of calls within an individual mixed trace. Unlike the former program, it produces highly complete reconstructions and, therefore, has a potential for wide application. The new method, implemented as a free web application Indelligent (http://ctap.inhs.uiuc.edu/dmitriev/indel.asp), employs dynamic programming optimization to estimate the pair of maximally similar strings that can be superimposed to produce the observed mixed sequence. We report the performance of the program on simulated mixed sequences, generated from pairs of superimposed strings containing single or multiple heterozygous indels and variable amounts of SNPs. We also describe results of validation tests, in which the program was used to decode 104 human traces, previously reported to contain heterozygous indels 5 to 30 bp [3], with a mean of 99.1% bases per allelic sequence reconstructed correctly and unambiguously. Additionally, we demonstrate that under ideal conditions the percentage of input ambiguous sites decoded by our program approaches 100%, which significantly exceeds the performance of ShiftDetector. Finally, we discuss limitations and potential applications of the new method.

## Materials and Methods

### Algorithms

#### Model and definitions

The essence of the problem is illustrated in Figure 1, which shows two allelic sequences, containing a heterozygous indel, properly aligned (A) and then misaligned due to removal of the gap (B). The consensus of the two misaligned strings (C), written using standard IUPAC symbols for degenerate nucleotide bases, represents all the information contained in the mixed trace (D). The goal, therefore, is to reconstruct (A) based on (C). At each ambiguous site, either of the two superimposed bases potentially can be placed into the upper or into the lower string. Because homologous allelic sequences are generally highly similar, our method arranges the superimposed bases in such a way as to obtain two strings with the maximum alignment score.

Let F = *a*_1_ *a*_2_…*a*_n_, be a string of letters representing successive peaks in a mixed trace. The letters representing superimposed identical peaks are A, C, G, or T, and other letters are IUPAC ambiguity symbols for 2-fold degenerate nucleotide bases: K, M, R, S, W, and Y (Figure 1, box). All the letters are called *bases*, and their positions 1≤*i*≤*n* are called *sites*. Define a *solution* as a pair of strings, the upper U = *u*_1_ *u*_2_…*u*_n_ and the lower L = *l*_1_ *l*_2_…*l*_n_, that contain no ambiguous bases and yield F if superimposed onto each other. Each ordered pair of bases 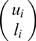 is called a *configuration*. If *a_i_* is an ambiguous base, the two corresponding alternative configurations are arbitrarily labeled with indexes *z_i_* = 1 and *z_i_* = 2. For example, for *a_i_* = R, 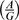 can be labeled as *z_i_* = 1 and 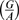 as *z_i_* = 2. For unambiguous bases the single possible configuration, where *u_i_* = *l_i_*, is labeled as *z_i_* = 1. The upper and the lower base in a configuration are denoted *u*(*i*, *z_i_*) and *l*(*i*, *z_i_*), respectively.

**Figure 3:**
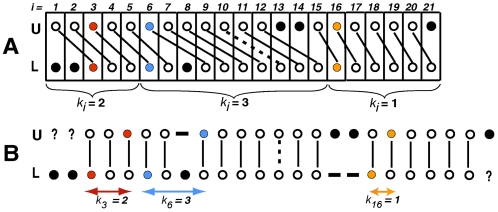
Two renderings of the same alignment, illustrating the concept of phase shift. Circles represent bases and links represent positional homologies: solid links–matches, and dashed links–mismatches. Black closed circles represent bases that have no positional homologs. In (A) vertical columns represent successive configurations of an aligned solution. The pairs of bases at sites 3, 6, and 16 are colored. Curly brackets mark segments aligned with different phase shifts. In (B) vertical columns contain pairs of positional homologs, and gaps are inserted opposite to bases having no homologs; external gaps are shown as question marks. The horizontal distances between the bases of each colored configuration (arrows) represent the corresponding phase shifts *k_i_*.

A solution in which pairwise relationships indicating positional homologies between bases of U and L have been established is called an *aligned solution*. Such a relationship between two identical bases is called a *match*, and between two different bases a *mismatch*. An aligned solution is diagrammatically illustrated by Figure 3A, in which vertical columns represent successive configurations at sites *i* from 1 to *n*. Figure 3B is a customary representation of the same alignment, where the vertical columns contain pairs of positional homologs, and gaps are inserted opposite to bases having no homologs. Observe that the two leftmost bases of L and one rightmost base in U can have homologs outside of F (Figure 3B, question marks). In contrast, the bases at site 8 in L, and sites 13 and 14 in U, according to this particular alignment, cannot have homologs. We use the term *inserted base* only for bases of the latter type. One or several consecutive inserted bases are called an *insertion*. For simplicity, we assume that all indels are insertions. For each *i* in an aligned solution (Figure 3A), we define *phase shift k_i_* as the horizontal distance between *u_i_* and *l_i_* after insertion of gaps (Figure 3B). If the mixed trace has resulted from a single insertion, at all *i* downstream of it, *k_i_* equals the number of inserted bases. In the more general case, *k_i_* is determined at each *i* by summation of all the insertions upstream of *i*. Insertions into opposite allelic strings can result in the mixed trace containing phase shifts of opposite directions. However, to simplify computation, we assume that all phase shifts have the same sign and that insertion of gaps always shifts *u_i_* right with respect to *l_i_* (Figure 3 A,B). The consequences of this simplification will be discussed below. In order to specify an aligned solution of F it is sufficient to specify values *z_i_* and *k_i_* for each *i*. A configuration for which the phase shift has been specified is called the *aligned configuration*, denoted *λ*(*i*, *z_i_*, *k*_i_).

#### Optimality criterion

We define the *alignment score* of an aligned solution as
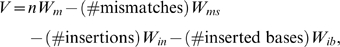
where *W_m_*, *W_ms_*, *W_i_*, and *W_ib_* are the weights of a match, mismatch, insertion, and inserted base, correspondingly. Multiplying *W_m_* by *n* instead of the actual number of matches, which would be more intuitive, makes it possible to obtain comparable scores at each site for every putative phase shift in our dynamic programming algorithm. The *optimal solution* is the one that can be aligned with the maximum *V*. Application of this criterion alone cannot guarantee biologically meaningful results for the following reasons. When the only indel is at the beginning or upstream of F, the solutions contain no insertions. Then the optimal solution is the one which has the minimum number of mismatches. For a given *n*, solutions with large *k_i_* always contain few mismatches simply because of the small overlap between the strings. Therefore, the chances of a solution being optimal due to pure chance increase as *k_i_*/*n* increases. On the other hand, consider a solution with an insertion of length *k_i_* and 0 mismatches and an alternative solution with a shorter insertion and *x*>0 mismatches. The first solution is better justified biologically because it explains F with fewer mutation events. However, because insertions are penalized in proportion to their length, the second solution can be optimal. It is easy to show that in this case, too, the chances of the second solution being optimal increase as *k_i_*/*n* increases. Therefore, to augment chances of selecting biologically meaningful optimal solutions, for each analyzed F we set an upper limit to the magnitude of putative phase shifts, denoted *K_max_*. Because our method relies exclusively on the information contained in the mixed trace, it is clear that reconstruction of a large heterozygous indel must require an adequately long input sequence. Our simulations, described below, have indicated that setting *K_max_* under *n*/10 is appropriate in many situations.

#### Computations

The goal is, therefore, to determine for each *i* from 1 to *n* such *λ*(*i*, *z_i_*, *k_i_*), where *z_i_* = 1 or 2 and 0≤*k_i_*≤*K_max_*, that the resulting solution has the maximum *V*. In order to do it, we estimate for each *λ*(*i*, *z_i_*, *k*_i_) the maximum *V* of all the solutions containing that configuration. For any *a_i_* in F consider strings F′ = *a*_1_ *a*_2_…*a_i_* and F″ = *a_i_a_i_*_+1_…*a_n_*. For each *λ*(*i*, *z_i_*, *k_i_*) denote the maximum *V* of all the aligned solutions of F′ that end with *λ*(*i*, *z_i_*, *k_i_*) as *P*(*i*, *z_i_*, *k_i_*). Denote the maximum *V* of all the aligned solutions of F″ that begin with *λ*(*i*, *z_i_*, *k_i_*) as *Q*(*i*, *z_i_*, *k_i_*). Let for a particular *λ*(*i*, *z_i_*, *k_i_*) the sum of the corresponding *P* and *Q* be the maximum of all aligned configurations at *i*. In that case, the maximum *V* of all the solutions of F containing *λ*(*i*, *z_i_*, *k_i_*) equals the maximum *V* of any solutions of F.

We use dynamic programming to compute for each *λ*(*i*, *z_i_*, *k*_i_), where *z_i_* = 1 or 2 and 0≤*k*_i_≤*K_max_*, estimates of the corresponding *P* and *Q*, denoted respectively *p*(*i*, *z_i_*, *k*_i_) and *q*(*i*, *z_i_*, *k*_i_). The difference of these scores from *P* and *Q* will be made clear below. Because estimation of *P* requires information only about sites 1 to *i* and that of *Q* requires information only about sites *i* to *n*, the matrix of *p*(*i*, *z_i_*, *k_i_*) is computed successively from *i* = 1 to *i* = *n*, and the matrix of *q*(*i*, *z_i_*, *k_i_*) in the opposite direction. Except for the execution order, the calculations are identical for both scores. Thus, only the computation of *p* is explained here in detail. The initial conditions follow from the definition of *V*:
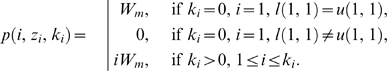



An insertion results in the magnitudes of phase shift being different between two successive sites (*k_i_*_−1_≠*k_i_*). Therefore, to accommodate potential insertions, for each *i*>*k_i_*, we first compute scores *p*′(*i*, *z_i_*, *k_i_*, *k_i_*_−1_), defined as estimates of the maximum score *V* of all the solutions of F′ which end with *λ*(*i*, *z_i_*, *k*_i_) and in which the preceding site is aligned with *k_i_*_−1_, where 0≤*k_i_*_−1_≤*K_max_*. Then *p*(*i*, *z_i_*, *k_i_*) is given by the maximum of these scores. Therefore, for *i*>*k_i_*,
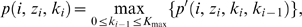
where
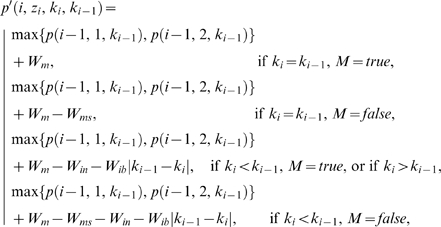
where
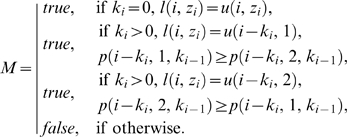
Each value *p*′(*i*, *z_i_*, *k_i_*, *k_i_*_−1_) is computed from the maximum value *p* obtained at the preceding site by adding *W_m_* and, if mismatches are introduced or a new phase shift is initiated, subtracting appropriate penalties as follows:

1. If *k_i_* = *k_i_*_−1_>0, *W_ms_* is subtracted when the aligned configuration *λ*(*i*, *z_i_*, *k*_i_) introduces a mismatch. This occurs when its lower base does not match the upper base of that configuration at the site *i*−*k_i_* which has yielded the maximum *p*(*i*−*k_i_*, *z_i_*, *k*_i_). This condition is identified above as *M* = *false*. Scoring for *k_i_* = *k_i_*_−1_ = 0 is straightforward.
2. If 0<*k_i_*<*k_i_*_−1_, *W_ms_* is subtracted when *λ*(*i*, *z_i_*, *k*_i_)introduces a mismatch, as explained above. Additionally, the affine penalty for insertion, *W_in_*+*W_ib_*|*k_i_*_−1_−*k_i_*|, is subtracted. Note that the number of inserted bases is given by the difference between the phase shift magnitudes.
3. If *k_i_*>*k_i_*_−1_, the affine penalty for insertion is subtracted as above. However, the penalty for mismatch is not subtracted even if the lower base of *λ*(*i*, *z_i_*, *k*_i_) introduces a mismatch. This point is explained by the following consideration. If a solution contains a transition from a smaller to a larger phase shift in the left to right direction, some bases in the lower string will be inserted bases (Figure 3). Scoring mismatches with such bases, which in fact have no homologs, will lead to spurious scores. We avoid this error by not evaluating the lower base of the configuration for mismatches in all cases when *k_i_*>*k_i_*_−1_ is hypothesized. As a result, both alternative configurations *z_i_* receive equal scores *p*. Therefore, for selected configurations, scores *p* may overestimate the true *P*. This approach may result in some of the sites remaining undecoded by the algorithm (for an additional mechanism attempting to reconstruct these sites see below), but not in errors. Computation of *q* includes a similar provision for the cases when *k_i_*>*k_i_*_+1_ is hypothesized, which allows to avoid scoring spurious mismatches with inserted bases in the upper string.

**Figure 4:**
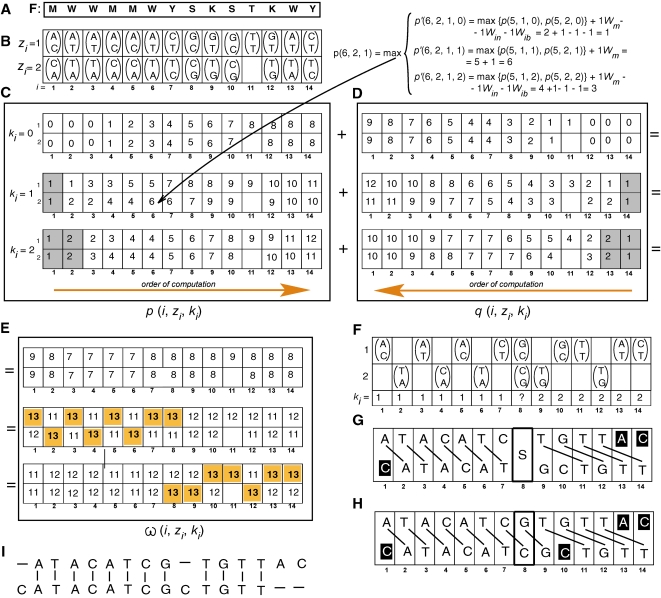
Main steps in decoding of a mixed trace. For the purpose of illustration, *K_max_* is set to 2, and the weights *W_m_*, *W_ms_*, *W_in_*, and *W_ib_* are all set to 1. Alternative configurations for each site of the input fragment F (A) are stored in the matrix (B). For each *k_i_* considered, 0, 1, and 2, a separate matrix is computed for *p* (C) and for *q* (D). Matrices for *k_i_*>0 are initialized with basal values at each *i*≤*k_i_*, shown in the grey cells. The remaining cells in the *p* matrices are filled out successively left to right and in the *q* matrices right to left. Each column has to be computed in all three matrices (one for each *k_i_*) before proceeding to the next site. For *i*>*k_i_*, computing each *p* and *q* requires first computing three *p*′ and *q*′ scores, correspondingly, one for each possible phase shift at the, respectively, preceding or following site. These calculations are omitted for space reasons, except for *p*(6, 2, 1), included as an example. The matrix of *ω*(*i*, *z_i_*, *k_i_*) is obtained by summation of *p* and *q* matrices; for each *i* the maximum values *ω* are highlighted (E). The configurations that received the maximum *ω*, and the corresponding *k_i_* are selected (F) to form the aligned solution (G). The site 7 remains ambiguous because both corresponding alternative configurations have yielded equal *ω*. The post-processing algorithm determines that only one of these can be incorporated without mismatches (H). The optimal aligned solution is output in the customary form (I). Symbols and conventions are as in Figure 3, except the bases having no homologs are shown on a black background.

For each *i*, the aligned configuration *λ*(*i*, *z_i_*, *k_i_*) which has yielded the maximum
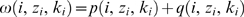
is selected to include in the estimate of the optimal solution. However, if *K_max_* is set too high, at a small number of consecutive sites, configurations aligned with large *k_i_* can receive maximum *ω* simply due to the large magnitude of the hypothesized phase shift (see above about the imperfection of the optimality criterion). These results in reconstructions containing a large insertion compensated for after just a few sites by an equally large deletion. To minimize the risk of this error, any *k_i_* which does not yield maximum *ω* in at least *k_i_*+1 consecutive sites is ignored, in which case the configurations yielding the next highest *ω* are selected. If two alternative configurations *z_i_* yield equal *ω*, with the same or with different phase shifts, the site remains ambiguous. This initial part of the decoding process is illustrated in Figure 4(A–G). The dynamic programming algorithm runs in space proportional to *n*(*K_max_*+1) and in time proportional to *n*(*K_max_*+1)^2^. The web server implementation of the algorithm requires approximately 3 sec to process a 500 bp input fragment when *K_max_* is set to 15 bp.

As a result of the conservative approach to scoring inserted bases, explained above, both alternative configurations in the vicinity of an indel may receive equal scores *ω*. An additional, post-processing algorithm attempts to reconstruct such ambiguous sites by considering consequences of each configuration being aligned with either of the two phase shifts reconstructed in the adjacent regions at the previous step. Aligned configurations that introduce minimum mismatches are incorporated in the output solution. For example, in Figure 4E both configurations at *i* = 8 have received equal *ω*. It is easy to see that only 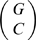 can be incorporated without creating a mismatch (Figure 4H), and therefore it is included in the final reconstruction (Figure 4I).

**Figure 5:**
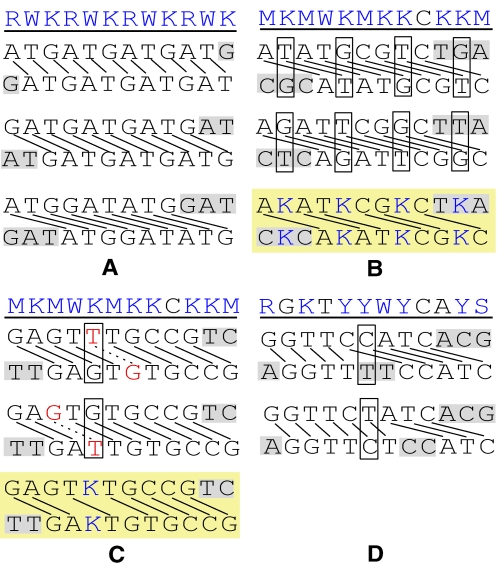
Examples of four main situations in which a mixed sequence fragment can have multiple cooptimal solutions. The fragments are shown on top of each panel, with their aligned optimal solutions and consensuses of these (on a yellow background) shown below. Solid links represent matches and dashed links mismatches, letters on a grey background represent bases with no positional homologs, blue letters represent ambiguous bases, and red letters mismatching bases. Configurations yielding equal maximum scores *ω* in (B), (C), and (D) are boxed. For the meanings of the IUPAC symbols see Figure 1. (A) A fully periodic fragment. Only three of 11 cooptimal solutions with different single phase shifts are shown. The consensus of these solutions is identical to the mixed fragment itself. (B) A fragment containing an ambiguous base (here “K”) repeated throughout the length of the fragment at regular intervals coinciding with the magnitude of the phase shift. The corresponding sites remain ambiguous in the consensus. Cooptimal solutions of this type are found mostly among fragments that are short with respect to the indel. (C) A fragment having cooptimal solutions with the same number but different locations of mismatches. Note that mismatches can either represent SNPs or result from basecalling errors. (D) A fragment containing an insertion that can be variably positioned. At one site, both alternative configurations yield maximum *ω*, each with a different phase shift.

When both alternative configurations yield optimal reconstructions, the site remains ambiguous in the output. This occurs when multiple cooptimal solutions of F objectively exist (Figure 5). In that case the output represents the consensus of cooptimal solutions.

At the sites in the vicinity of an indel the same *z_i_* can yield the maximum *ω* when aligned with two different phase shifts *k_i_*. This occurs when multiple alignments of the same optimal solution, with alternative placements of the gap, are possible. Such floating gaps occur when an insertion begins or ends with the base identical to the base following or preceding the insertion, respectively. In these cases, the most likely position of the gap can sometimes be determined from structural considerations [17].

**Figure 6:**
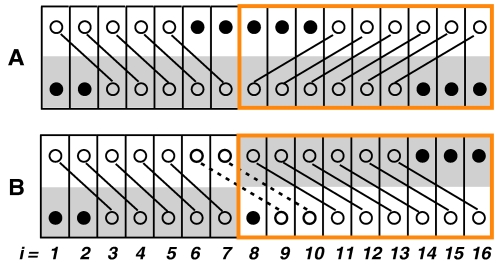
Two aligned solutions of the same mixed fragment, representing the transition between the phase shifts *k_i_* = 2 and *k_i_* = 3, alternatively, as a “long” insertion (A) or a “short” insertion (B). Bases corresponding to the lower allelic string in (A) are highlighted with grey. Note that one solution can be obtained from another by swapping the parts of the allelic strings between sites 8 and 16 (orange box).

Decoding mixed traces resulting from multiple indels presents a special difficulty because any change in the phase shift, except changes from or to *k_i_* = 0, can be explained alternatively by a short or a long insertion placed in the opposite strings (Figure 6A and 6B). Either variant can be optimal, depending on the weighting scheme. However, because the scores are computed under the assumption that all phase shifts have the same direction, only the short variant is reconstructed (Figure 6B). For a practical solution, which allows to visualize alternative reconstructions of an indel, see the next section. The problem can be avoided by analyzing both direct and reverse sequences of the same template. Note that decoding of sequences resulting from a single indel does not involve an uncertainty of this kind.

In practice, traces occasionally contain sites with more than two superimposed peaks. Therefore, we have modified the above algorithm to additionally handle IUPAC symbols for 3-fold degenerate bases (B, H, D, V) and unknown bases (N). For a site containing one of these symbols, a single configuration, where *u_i_* = *l_i_*, is considered. Therefore, the site remains ambiguous until the alignment of the optimal solution is produced. Then, if two homologous symbols represent, respectively, sets of bases *X* and *Y*, the intersection *X* ∩ *Y* is written into both strings.

### Implementation

The method has been implemented as a free web application Indelligent (http://ctap.inhs.uiuc.edu/dmitriev/indel.asp). The program takes as input a sequence of IUPAC symbols representing non-degenerate and degenerate nucleotide bases. Such sequences are output by customary autosequencer software, such as PHRED [18] or KBBasecaller (Applied Biosystems). In the trace files output by other basecallers, the sites containing superimposed peaks can be recalled with IUPAC symbols using Sequencher (Gene Codes Corp., Ann Arbor, MI). The default weighting scheme is *W_m_*, *W_ms_*, *W_ib_* = 1, and *W_in_* = 2. Because of the prevalence of short indels, in order to speed up computations, the default *K_max_* is 15 bp. The user can change *W_in_* to any positive integer, and *K_max_* to any positive integer up to half length of the input sequence. The program outputs a pair of aligned reconstructed allelic sequences. Floating gaps can be aligned, alternatively, left or right. Additionally, the *Display “long” indels* option swaps parts of the predicted allelic sequences to display the alternative, long reconstruction of the indel (Figure 6). The source code, free for non-commercial users, is available at the Indelligent web site.

### Validation tests

#### Simulations

Pairs of identical strings composed of random bases A, C, G, and T, selected with equal probability, were generated and shifted with respect to each other by inserting additional bases into one or both strings. To simulate single nucleotide polymorphisms (SNPs), point differences between the strings were introduced at randomly chosen sites in the overlapping parts of the strings. The consensus of the strings, except the overhanging parts at the beginning and end, was input for analysis. For each combination of tested parameters (see below) 1,000 sequence fragments were generated and analyzed with the default weighting scheme. For each fragment, the upper output string was compared to the upper generated string and the number of positions reconstructed correctly and unambiguously, ambiguously, or incorrectly, was recorded. We also recorded whether the correct phase shifts were detected. Three sets of experiments were conducted:

*One phase shift: 5 bp*. 50, 75, and 100 bp fragments were generated from string pairs containing an extra 5 bp at the origin of one string. This corresponds to the practical situation when one attempts to unscramble the mixed trace downstream of a single insertion. Seven levels of divergence between alleles, from 0 to 20%, were set by varying the number of point differences; exact divergencies varied between lengths. The maximum divergence tested exceeded the record average nucleotide heterozygosity observed in nature [19] more than four times. The analyses were run with *K_max_* = 15 bp.

*Two phase shifts: 0 bp and x>0 bp*. Because, in real applications, a mixed trace usually follows an unambiguous trace (Figure 1), we simulated fragments containing such a transition. 100 bp fragments were generated from string pairs containing a single insertion of 1, 7, 10, 12, or 14 bp in the middle of one string. The tested size range accommodated the vast majority of indel sizes encountered in nature [19]–[24]. The analyses were run with *K_max_* = 25 bp.

*Two phase shifts: x, y>0 bp*. To assess how the program handles mixed fragments resulting from multiple indels, 100 bp fragments were generated from string pairs containing an extra 3, 5, or 8 bp at the origin of one string and an 8 bp insertion in the middle of the same or the opposite strings. No SNPs were simulated in this experiment. The analyses were run with *K_max_* = 25.

#### Human traces

198 mixed human traces in which Bhangale et al. [3] discovered heterozygous indels between 5 and 30 bp, were obtained from NCBI Trace Archive (http://www.ncbi.nlm.nih.gov/Traces/trace.cgi). Because large indels are harder to reconstruct, we did not test the program on the traces containing short indels, reported in the same study. The Sequencher Ver 4.6 (Gene Codes Corp., Ann Arbor, MI) program was used to call secondary peaks at least 20% as high as the primary peaks in each trace. The traces were then inspected and basecalling errors were corrected manually to the degree possible. For each trace, the fragment to analyze was determined as follows. Because in areas of repeated sequence the entire indel or a part of it can be located upstream of the first double peak observed, 100 sites upstream of the first double peak or as many as available were included. To account for deterioration of signal toward the end of each trace, we marked the position where the first 3-fold degenerate site was encountered that could not be confidently recoded as a 2-fold degenerate site as the end of the potentially interpretable fragment. Traces of low quality and those yielding less than 100 bp of potentially interpretable mixed trace were excluded. The remaining 104 fragments, containing 103–677 bp (mean 294±126, SD) of mixed trace, were analyzed with *K_max_* = 30 bp, *W_m_*, *W_ms_*, *W_ib_* = 1, and *W_in_* = 2. Floating gaps were aligned in the extreme left position. For each fragment analyzed, the reconstructed insertion plus the strict consensus of two aligned reconstructed allelic sequences downstream of it were aligned with 50 best matching human sequences in NCBI Trace Archive database using BLASTN 2.2.17 (http://www.ncbi.nlm.nih.gov/BLAST/) [25] with default parameters and no filtering of low-complexity regions. We assumed that most polymorphisms in the analyzed mixed traces must be represented among sequences in the database. Therefore, an unambiguously reconstructed site was scored as an error if none of the matching database sequences contained the same base, and as correct otherwise. An ambiguous site was scored as a putative SNP if it represented two bases and each was present in at least one of the database sequences, as an error if neither was present, and as ambiguous otherwise. Finally, we reexamined the traces to determine whether the erroneous reconstructions have resulted from basecalling errors.

### Indelligent *vs.* ShiftDetector

We used human traces also to compare the proportions of ambiguous sites decoded by ShiftDetector [15] and by Indelligent. Because the first program takes as input raw chromatogram files and the second takes sequences, in order to minimize the effect of this difference on the results we manually selected among the trace files listed by Bhangale et al. [3] 55 chromatograms containing 4 or 5 bp heterozygous indels, each with at least 100 bp of high-quality mixed trace (*i.e.*, with primary and secondary peaks well aligned, and no tertiary peaks or background noise). For details on the chromatograms see Table S5. Secondary peaks were called using Sequencher as described above, and the resulting sequences were input to Indelligent without prior editing. The raw files were processed with ShiftDetector. For each trace, we determined the number of ambiguous sites in the first 100 bp following the indel, as decoded by each program. For Indelligent, we scored ambiguities in the strict consensus of two reconstructed allelic sequences, which is equivalent to the single sequence output by ShiftDetector.

## Results

**Figure 7:**
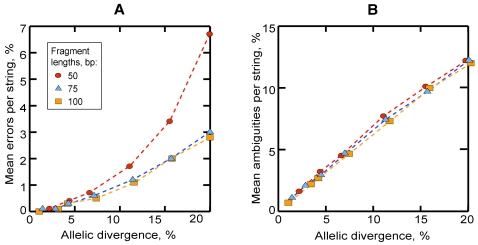
Accuracy of decoding of simulated mixed fragments formed by 5 bp shift at the origin of one of two allelic strings. The horizontal axis represents divergence between the allelic strings forming each fragment. (A) Mean percent of erroneous bases per reconstructed string. (B) Mean percent of ambiguous bases per reconstructed string. Each point represents the mean of 1,000 runs. For SD and additional statistics, see Table S1.

In the experiments on simulated fragments with a single 5 bp phase shift, the proportion of 50 bp fragments reconstructed with the single correct phase shifts was 100% up to 6.7% divergence and progressively decreased at larger divergences. For both 75 bp and 100 bp fragments, no false phase shifts were found up to 11.4% and 15.8% divergencies, respectively. For divergencies up to 4%, the mean number of incorrect bases per decoded string was 0.1–0.4% (SD<0.7%) for all fragment lengths tested (Figure 7A). The proportion of errors increased with increasing divergence. In 50 bp fragments it grew markedly faster after ca. 10% divergence as a consequence of the increased number of reconstructed false phase shifts 10–15 bp long. The mean proportion of ambiguous bases per reconstructed string increased as approximately 0.7(*divergence between alleles*) regardless of the length of the fragment (Figure 7B). For detailed results see Table S1.

**Figure 8:**
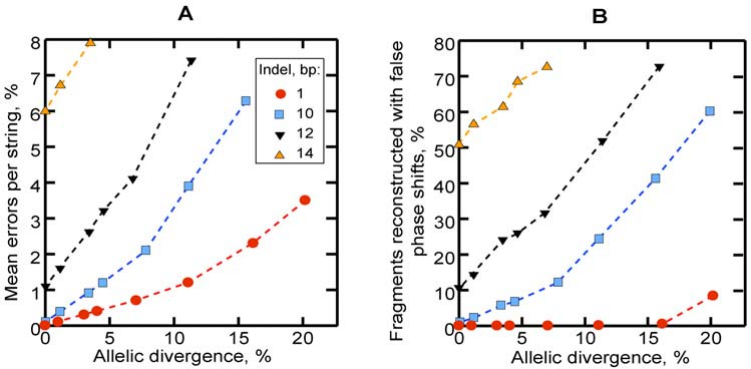
Accuracy of decoding of simulated 100 bp mixed fragments containing a single insertion of variable size in the middle. (A) Mean percent of erroneous bases per reconstructed string. (B) Percent of fragments reconstructed with incorrect indels. Each point represents 1,000 runs. For SD and additional statistics, see Table S2.

In the tests simulating a transition between the unambiguous and mixed parts of a trace, the accuracy of reconstruction dropped sharply for indels above 10 bp (Figure 8A) due to the increased number of fragments reconstructed with incorrect, shorter phase shifts (Figure 8B). For smaller indels, the mean number of errors did not exceed 1.2% per string for allelic divergencies up to 4.4% (Table S2).

100 bp fragments resulting from two indels were decoded with a mean of 1.4% of errors per string or less, except in the experiment with a 8 bp shift at the origin and additional 8 bp inserted in the middle of the same string, in which the accuracy of decoding was lower (Table S3).

102 human mixed sequences were reconstructed with a single indel and two sequences with two indels. 67 fragments were reconstructed without errors, 31 with 1–2 errors, and six with 3–7 errors. The mean number of erroneously reconstructed bases per fragment was 0.66 (SD = 1.21). Because no correlation was found between the fragment length and the number of errors (P = 0.572), the error rate per base is not reported. Half of the fragments were reconstructed without ambiguities and half with 1–10 ambiguous bases. The mean number of bases reconstructed correctly and unambiguously per fragment was 99.1% (SD = 1.25, minimum 92.5, median 99.6). Reexamination of traces revealed that at least 41 (59.4%) errors resulted from incorrect basecalling, mostly in low-quality trace regions. Details on the results are given in Table S4.

In our comparisons, Indelligent decoded 92.8 to 100.0% of ambiguous sites (mean 98.9±1.95%, SD, median 100%), while ShiftDetector decoded only 56.0 to 85.5% of ambiguous sites (mean 72.5±6.47%, SD, median 73.0%). The details are given in Table S5.

## Discussion

We have demonstrated that an individual mixed trace formed by single or multiple heterozygous indels can be deconvoluted with a high degree of accuracy in the absence of additional information. Because the method estimates optimal solutions as pairs of maximally similar strings, it is expected to produce errors in proportion to the degree of divergence between the superimposed allelic sequences (Figure 7A and 8A). While errors can occur even if phase shifts are determined correctly (when the optimal solution contains less mismatches than was actually present between the superimposed allelic sequences), their number increases dramatically when false phase shifts are found. To minimize the chance of reconstructing false phase shifts, *K_max_* should be set appropriately low with respect to the fragment length. In experiments with the 100 bp fragments simulating a transition between unambiguous and mixed traces, the accuracy dropped for *k_i_*>10 bp (Figure 8A). For larger indels, both lowering *K_max_* or increasing the length of the analyzed fragment can improve results.

Experiments with fragments of variable length demonstrated that, if *K_max_* is set appropriately, the accuracy is similar for different fragment lengths (75 and 100 bp fragments, Figure 7A and 7B). Therefore, although our simulation tests were conducted on relatively short fragments, the results can be extrapolated to longer sequences.

The highest accuracy of reconstruction is achieved when the analyzed fragment is significantly larger than the indel (ca. 10 times with the default weighting scheme) and when it is formed by highly similar allelic sequences. These conditions occur in the vast majority of cases when heterozygous indels are encountered. Indels between 1 and 10 bp in size account for the majority of indels in the genomes of man (92.3%, calculated from data in [24]) and other eukaryotes [19]–[23]. Therefore, in most cases the length of the available mixed sequence is sufficient for its decoding. The average divergence between two sequences randomly drawn from a population does not exceed 0.1% for human noncoding DNA [26], 1–2% for fruit fly noncoding DNA [27], with the overall record of 4.5% measured for the genomic DNA of sea squirts [19]. Our simulations indicated that, within this range of within-individual allelic divergence, the average number of erroneously reconstructed bases per fragment is expected to be between 0 and 1.2% if the weighting scheme and *K_max_* are properly chosen. In practice, however, the accuracy of reconstruction is affected by basecalling errors, which are particularly frequent in calling superimposed peaks. In our tests, reexamination of the human traces revealed that ca. 60% of the erroneously reconstructed sites were due to basecalling errors missed during the initial inspection of the recalled trace. Other base predictions scored as errors are likely to represent rare polymorphisms absent in the database. Therefore, the reported mean of 0.66 errors per reconstructed fragment must considerably underestimate the potential accuracy of the method.

The method is capable of reconstructing mixed traces resulting from multiple indels. Yet, when the distance between two adjacent indels is small, the cost of an additional insertion can be higher than the cost of mismatches in the alternative solution. Therefore, the success of decoding depends on the particular weighting scheme and how widely the adjacent indels are spaced. Failed reconstructions generally result in a large number of mismatches and ambiguities in the output. This allows adjusting parameters iteratively until a satisfactory reconstruction is obtained. Incorporating a test of the statistical significance of reconstructed optimal solutions in the future would give the method additional robustness.

Indelligent outperforms ShiftDetector [15] by producing a complete, biallelic reconstruction for the entire input fragment, including single or multiple indels. Even more importantly, it decodes all or nearly all input ambiguous sites, extracting all the information that can be extracted from an individual mixed trace. The method can find application in all situations where mixed traces formed by heterozygous indels are encountered, including situations where neither a suitable reference, nor a reverse trace are available, or when speed is crucial. It can be easily bundled with tools for chromatogram processing, sequence editing, and mutation discovery. In addition to applications aimed at detection and characterization of nucleotide polymorphisms, unscrambling of mixed traces is crucial in situations where the obscured sequence downstream of the indel is of primary interest. In particular, the new method can be used in the molecular phylogenetic studies of introns and intergenic regions, which provide fast-evolving nuclear markers for estimating relationships between recently diverged taxa, but often are hard to sequence directly because of the high frequency of indels [28]. In such projects, the method can serve as a cost-efficient alternative to expensive cloning. For example, we successfully used Indelligent to decode mixed traces obtained by direct sequencing of an indel-rich intron region of the elongation factor-1 alpha gene for a phylogenetic study of the little-studied leafhopper genus *Cuerna* (Insecta, Hemiptera, Cicadellidae), for which no sequences were available to use as a reference. The results of this study will be published separately.

## Supporting Information

Table S1 Accuracy of decoding of simulated mixed fragments formed by 5 bp shift at the origin of one of two allelic strings. Each row summarizes analyses of 1,000 fragments. For details on the experiments see Materials and Methods.
(0.06 MB DOC)

Table S2 Accuracy of decoding of simulated mixed 100 bp fragments formed by inserting variable number of bases in the middle of one of two allelic strings. Each row summarizes analyses of 1,000 fragments. For details on the experiments see Materials and Methods.
(0.09 MB DOC)

Table S3 Accuracy of decoding of simulated 100 bp fragments resulted from two indel events: shifting the origin of one of two allelic strings x bp and insertion of y bp in the middle of the same (location indicated as “+”) or the opposite (“−”) strings. Each row summarizes analyses of 1,000 fragments. For details on the experiments see Materials and Methods.
(0.04 MB DOC)

Table S4 Decoded mixed human traces, and the number of errors, putative SNPs, and other ambiguous bases in the consensus reconstructions as revealed by BLASTN comparisons with sequences in the NCBI Trace Archive database.
(0.21 MB DOC)

Table S5 Completeness of reconstruction by ShiftDetector and by Indelligent, measured as the percentage of the input ambiguous sites decoded by each program.
(0.08 MB DOC)
