# Supplementary material for: Decoding of Superimposed Traces Produced by Direct Sequencing of Heterozygous Indels
Source: PLoS Comput Biol. 2008 Jul 25;4(7):e1000113. doi: 10.1371/journal.pcbi.1000113 (PMC2429969; doi:10.1371/journal.pcbi.1000113)
Supplement: Table S1 — Accuracy of decoding of simulated mixed fragments formed by 5 bp shift at the origin of one of two allelic strings. Each row summarizes analyses of 1,000 fragments. For details on the experiments see Materials and Methods. (0.06 MB DOC) [file pcbi.1000113.s001.doc]

**Table S1.** Accuracy of decoding of simulated mixed fragments formed by 5 bp shift at the origin of one of two allelic strings. Each row summarizes analyses of 1,000 fragments. For details on the experiments see Materials and Methods.

| Fragment length, bp | Divergence between  alleles,  % | Mean correct & unambiguous bases per decoded string,  % ± SD | Mean errors per decoded string,  % ± SD | Fragments decoded with 0 errors,  % | Fragments decoded with <2 errors per string,  % | Fragments decoded with <3 errors per string,  % | Median errors per decoded  string,  % | Maximum errors  per decoded  string,  % | Mean ambiguities per decoded  string,  % ± SD | Fragments decoded  with false indels,  % |
| --- | --- | --- | --- | --- | --- | --- | --- | --- | --- | --- |
| 50 | 0.00 | 100.0 ± 0.00 | 0.0 ± 0.00 | 100.0 | 100.0 | 100.0 | 0.0 | 0.0 | 0.0 ± 0.00 | 0.0 |
| 50 | 2.22 | 98.3 ± 2.38 | 0.1 ± 0.35 | 96.3 | 98.4 | 99.4 | 0.0 | 8.0 | 1.6 ± 2.36 | 0.0 |
| 50 | 4.44 | 96.4 ± 3.00 | 0.4 ± 0.63 | 87.9 | 94.5 | 98.3 | 0.0 | 10.0 | 3.2 ± 3.00 | 0.0 |
| 50 | 6.67 | 94.8 ± 3.87 | 0.7 ± 0.81 | 78.4 | 91.1 | 97.2 | 0.0 | 16.0 | 4.5 ± 3.94 | 0.0 |
| 50 | 11.11 | 90.6 ± 5.47 | 1.7 ± 1.51 | 59.5 | 79.4 | 90.4 | 0.0 | 22.0 | 7.7 ± 5.06 | 1.9 |
| 50 | 15.56 | 86.5 ± 7.81 | 3.4 ± 2.46 | 40.7 | 61.5 | 78.4 | 2.0 | 32.0 | 10.1 ± 6.30 | 7.8 |
| 50 | 20.00 | 81.1 ± 10.68 | 6.7 ± 3.84 | 23.1 | 42.7 | 58.0 | 4.0 | 40.0 | 12.2 ± 7.52 | 23.7 |
| 75 | 0.00 | 100.0 ± 0.00 | 0.0 ± 0.00 | 100.0 | 100.0 | 100.0 | 0.0 | 0.0 | 0.0 ± 0.00 | 0.0 |
| 75 | 1.43 | 98.9 ± 1.44 | 0.1 ± 0.29 | 97.6 | 98.6 | 99.8 | 0.0 | 5.3 | 1.1 ± 1.43 | 0.0 |
| 75 | 2.86 | 97.8 ± 2.09 | 0.1 ± 0.43 | 93.1 | 97.3 | 99.3 | 0.0 | 5.3 | 2.1 ± 2.09 | 0.0 |
| 75 | 4.29 | 96.8 ± 2.46 | 0.3 ± 0.67 | 86.7 | 92.9 | 97.8 | 0.0 | 6.7 | 2.9 ± 2.45 | 0.0 |
| 75 | 7.14 | 94.8 ± 3.23 | 0.6 ± 0.87 | 74.8 | 87.2 | 96.0 | 0.0 | 9.3 | 4.7 ± 3.27 | 0.0 |
| 75 | 11.43 | 91.4 ± 3.97 | 1.2 ± 1.28 | 52.3 | 73.9 | 89.2 | 0.0 | 9.3 | 7.4 ± 4.08 | 0.2 |
| 75 | 15.71 | 88.4 ± 4.58 | 2.0 ± 1.52 | 33.4 | 58.9 | 78.7 | 1.3 | 12.0 | 9.7 ± 4.61 | 0.2 |
| 75 | 20.00 | 84.8 ± 5.36 | 3.0 ± 2.07 | 22.0 | 43.1 | 62.6 | 2.7 | 22.7 | 12.2 ± 5.36 | 2.2 |
| 100 | 0.00 | 100.0 ± 0.00 | 0.0 ± 0.00 | 100.0 | 100.0 | 100.0 | 0.0 | 0.0 | 0.0 ± 0.00 | 0.0 |
| 100 | 1.05 | 99.3 ± 0.99 | 0.0 ± 0.29 | 97.5 | 98.9 | 99.5 | 0.0 | 3.0 | 0.7 ± 0.98 | 0.0 |
| 100 | 3.16 | 97.7 ± 1.85 | 0.1 ± 0.51 | 91.7 | 96.6 | 98.8 | 0.0 | 5.0 | 2.2 ± 1.84 | 0.0 |
| 100 | 4.21 | 97.0 ± 2.04 | 0.3 ± 0.72 | 83.9 | 92.6 | 97.5 | 0.0 | 6.0 | 2.7 ± 2.02 | 0.0 |
| 100 | 7.37 | 94.6 ± 2.75 | 0.5 ± 0.98 | 69.2 | 84.7 | 94.1 | 0.0 | 6.0 | 4.8 ± 2.8 | 0.0 |
| 100 | 11.58 | 91.5 ± 3.36 | 1.1 ± 1.36 | 45.9 | 70.2 | 85.4 | 1.0 | 9.0 | 7.4 ± 3.42 | 0.0 |
| 100 | 15.79 | 88.1 ± 4.25 | 2.0 ± 1.75 | 23.7 | 46.7 | 67.5 | 2.0 | 12.0 | 10 ± 4.29 | 0.2 |
| 100 | 20.00 | 85.2 ± 4.35 | 2.8 ± 2.1 | 11.0 | 30.2 | 50.5 | 2.0 | 12.0 | 12 ± 4.57 | 1.1 |
