# Supplementary material for: Decoding of Superimposed Traces Produced by Direct Sequencing of Heterozygous Indels
Source: PLoS Comput Biol. 2008 Jul 25;4(7):e1000113. doi: 10.1371/journal.pcbi.1000113 (PMC2429969; doi:10.1371/journal.pcbi.1000113)
Supplement: Table S2 — Accuracy of decoding of simulated mixed 100 bp fragments formed by inserting variable number of bases in the middle of one of two allelic strings. Each row summarizes analyses of 1,000 fragments. For details on the experiments see Materials and Methods. (0.09 MB DOC) [file pcbi.1000113.s002.doc]

**Table S2.** Accuracy of decoding of simulated mixed 100 bp fragments formed by inserting variable number of bases in the middle of one of two allelic strings. Each row summarizes analyses of 1,000 fragments. For details on the experiments see Materials and Methods.

| Indel length, bp | Divergenvce between alleles,  % | Mean correct & unambiguous bases per decoded string,  % ± SD | Mean errors per decoded  string,  % ± SD | Fragments decoded with 0 errors,  % | Fragments decoded with <2 errors per string,  % | Fragments decoded with <3 errors per string,  % | Median errors per decoded  string, % | Maximum errors  per decoded  string,  % | Mean ambiguities per decoded string,  % ± SD | Fragments decoded with false indels,  % |
| --- | --- | --- | --- | --- | --- | --- | --- | --- | --- | --- |
| 1 | 0.00 | 100.0 ± 0.00 | 0.0 ± 0.00 | 100.0 | 100.0 | 100.0 | 0.0 | 0 | 0.0 ± 0.00 | 0.0 |
| 1 | 1.01 | 99.2 ± 0.72 | 0.1 ± 0.26 | 93.4 | 99.8 | 100.0 | 0.0 | 2 | 0.7 ± 0.74 | 0.0 |
| 1 | 3.03 | 97.7 ± 1.22 | 0.3 ± 0.57 | 77.2 | 96.8 | 99.5 | 0.0 | 6 | 2.1 ± 1.26 | 0.0 |
| 1 | 4.04 | 96.9 ± 1.38 | 0.4 ± 0.67 | 67.8 | 94.5 | 98.8 | 0.0 | 5 | 2.7 ± 1.41 | 0.0 |
| 1 | 7.07 | 94.5 ± 1.87 | 0.7 ± 0.95 | 50.0 | 83.3 | 94.7 | 0.5 | 7 | 4.8 ± 1.93 | 0.0 |
| 1 | 11.11 | 91.3 ± 2.32 | 1.2 ± 1.27 | 33.3 | 65.7 | 85.4 | 1.0 | 8 | 7.4 ± 2.38 | 0.1 |
| 1 | 16.16 | 87.5 ± 2.65 | 2.3 ± 1.77 | 12.8 | 36.4 | 59.8 | 2.0 | 14 | 10.2 ± 2.86 | 0.5 |
| 1 | 20.20 | 84.2 ± 3.34 | 3.5 ± 2.77 | 6.5 | 21.8 | 41.2 | 3.0 | 23 | 12.3 ± 3.30 | 8.4 |
| 7 | 0.00 | 100.0 ± 0.13 | 0.0 ± 0.00 | 100.0 | 100.0 | 100.0 | 0.0 | 0 | 0.0 ± 0.13 | 0.0 |
| 7 | 1.08 | 99.2 ± 0.76 | 0.2 ± 0.52 | 84.0 | 97.3 | 98.9 | 0.0 | 4 | 0.6 ± 0.64 | 0.7 |
| 7 | 3.23 | 97.6 ± 1.19 | 0.6 ± 0.88 | 57.0 | 86.0 | 96.3 | 0.0 | 6 | 1.8 ± 1.05 | 2.3 |
| 7 | 4.30 | 96.8 ± 1.48 | 0.8 ± 1.05 | 50.1 | 81.8 | 93.3 | 0.0 | 8 | 2.4 ± 1.30 | 3.2 |
| 7 | 7.53 | 94.3 ± 1.86 | 1.5 ± 1.46 | 28.1 | 59.9 | 80.7 | 1.0 | 10 | 4.2 ± 1.64 | 7.0 |
| 7 | 11.83 | 91.1 ± 2.46 | 2.6 ± 1.98 | 11.5 | 33.8 | 56.1 | 2.0 | 12 | 6.3 ± 2.06 | 14.3 |
| 7 | 16.13 | 87.4 ± 3.94 | 4.0 ± 2.92 | 4.1 | 16.3 | 33.5 | 3.0 | 22 | 8.6 ± 3.21 | 23.4 |
| 7 | 20.43 | 82.0 ± 8.10 | 6.4 ± 5.08 | 4.3 | 9.5 | 19.2 | 5.0 | 28 | 11.6 ± 7.48 | 37.6 |
| 10 | 0.00 | 99.7 ± 1.45 | 0.1 ± 0.65 | 98.9 | 99.0 | 99.2 | 0.0 | 13 | 0.2 ± 1.09 | 1.0 |
| 10 | 1.11 | 98.9 ± 1.99 | 0.4 ± 1.24 | 79.3 | 96.1 | 97.5 | 0.0 | 22 | 0.8 ± 1.25 | 1.8 |
| 10 | 3.33 | 97.2 ± 2.25 | 0.9 ± 1.59 | 53.2 | 80.8 | 92.0 | 0.0 | 19 | 1.9 ± 1.44 | 5.7 |
| 10 | 4.44 | 96.2 ± 2.51 | 1.2 ± 1.73 | 39.0 | 69.8 | 87.2 | 1.0 | 19 | 2.5 ± 1.76 | 6.6 |
| 10 | 7.78 | 93.6 ± 3.42 | 2.1 ± 2.60 | 19.5 | 49.6 | 70.8 | 2.0 | 24 | 4.2 ± 1.89 | 12.1 |
| 10 | 11.11 | 90.0 ± 5.38 | 3.9 ± 4.02 | 8.0 | 22.6 | 45.5 | 3.0 | 26 | 6.1 ± 2.79 | 24.4 |
| 10 | 15.56 | 84.3 ± 8.95 | 6.3 ± 5.34 | 4.8 | 12.0 | 23.0 | 5.0 | 29 | 9.5 ± 7.38 | 41.4 |
| 10 | 20.00 | 76.8 ± 11.51 | 8.8 ± 6.46 | 10.4 | 12.0 | 16.2 | 7.0 | 27 | 14.4 ± 11.94 | 60.1 |
| 12 | 0.00 | 97.9 ± 5.08 | 1.1 ± 3.43 | 87.3 | 88.3 | 89.2 | 0.0 | 22 | 1.0 ± 2.18 | 10.6 |
| 12 | 1.14 | 96.6 ± 5.90 | 1.6 ± 4.01 | 68.3 | 82.4 | 85.2 | 0.0 | 23 | 1.7 ± 2.47 | 14.3 |
| 12 | 3.41 | 94.4 ± 6.52 | 2.6 ± 4.51 | 38.3 | 63.9 | 75.2 | 1.0 | 25 | 3.0 ± 2.96 | 23.8 |
| 12 | 4.55 | 93.1 ± 7.08 | 3.2 ± 4.73 | 28.7 | 53.5 | 69.0 | 1.0 | 31 | 3.7 ± 3.49 | 26.0 |
| 12 | 6.82 | 90.7 ± 7.91 | 4.1 ± 5.37 | 16.8 | 40.4 | 59.0 | 2.0 | 29 | 5.1 ± 4.23 | 31.5 |
| 12 | 11.36 | 84.4 ± 9.79 | 7.4 ± 6.58 | 6.5 | 16.6 | 29.6 | 5.0 | 26 | 8.2 ± 5.93 | 51.8 |
| 12 | 15.91 | 77.0 ± 10.50 | 10.3 ± 6.68 | 6.8 | 9.1 | 14.3 | 10.0 | 27 | 12.8 ± 9.55 | 72.8 |
| 12 | 20.45 | 71.0 ± 10.98 | 11.5 ± 7.20 | 14.8 | 15.5 | 17.1 | 13.0 | 28 | 17.5 ± 13.32 | 84.5 |
| 14 | 0.00 | 90.1 ± 10.07 | 6.0 ± 7.15 | 46.3 | 48.1 | 50.3 | 2.0 | 28 | 3.9 ± 4.08 | 50.8 |
| 14 | 1.16 | 88.5 ± 10.22 | 6.7 ± 7.13 | 32.7 | 41.5 | 45.4 | 4.0 | 24 | 4.8 ± 4.56 | 56.5 |
| 14 | 3.50 | 85.9 ± 10.25 | 7.9 ± 7.24 | 19.6 | 32.4 | 39.2 | 6.0 | 26 | 6.2 ± 5.21 | 61.4 |
| 14 | 4.65 | 83.9 ± 10.10 | 9.0 ± 7.02 | 46.3 | 48.1 | 50.3 | 9.0 | 26 | 7.1 ± 5.16 | 50.8 |
| 14 | 6.98 | 81.3 ± 10.17 | 10.2 ± 7.06 | 32.7 | 41.5 | 45.4 | 11.0 | 29 | 8.5 ± 5.93 | 56.5 |
| 14 | 11.63 | 76.2 ± 9.42 | 12.0 ± 6.39 | 19.6 | 32.4 | 39.2 | 14.0 | 29 | 11.8 ± 8.28 | 61.4 |
| 14 | 15.12 | 73.8 ± 9.70 | 12.2 ± 6.58 | 12.8 | 24.3 | 30.6 | 13.0 | 29 | 14 ± 10.27 | 68.5 |
| 14 | 19.77 | 70.4 ± 9.90 | 12.2 ± 6.95 | 7.6 | 17.0 | 23.8 | 14.0 | 30 | 17.4 ± 12.52 | 72.6 |
