# Supplementary material for: Decoding of Superimposed Traces Produced by Direct Sequencing of Heterozygous Indels
Source: PLoS Comput Biol. 2008 Jul 25;4(7):e1000113. doi: 10.1371/journal.pcbi.1000113 (PMC2429969; doi:10.1371/journal.pcbi.1000113)
Supplement: Table S3 — Accuracy of decoding of simulated 100 bp fragments resulted from two indel events: shifting the origin of one of two allelic strings x bp and insertion of y bp in the middle of the same (location indicated as “+”) or the opposite (“−”) strings. Each row summarizes analyses of 1,000 fragments. For details on the experiments see Materials and Methods. (0.04 MB DOC) [file pcbi.1000113.s003.doc]

**Table S3.** Accuracy of decoding of simulated 100 bp fragments resulted from two indel events: shifting the origin of one of two allelic strings *x* bp and insertion of *y* bp in the middle of the same (location indicated as “+”) or the opposite (“−”) strings. Each row summarizes analyses of 1,000 fragments. For details on the experiments see Materials and Methods.

| *x*, bp | Location of insertion | *y*, bp | Mean correct & unambiguous bases per decoded string,  % ± SD | Mean errors  per decoded string,  % ± SD | Fragments decoded with 0 errors,  % | Fragments decoded with <2 errors per string,  % | Fragments decoded with <3 errors per string,  % | Median errors per decoded  string,  % | Maximum errors per decoded  string,  % | Mean ambiguities  per decoded  string,  % ± SD | Fragments decoded  with false indels,  % |
| --- | --- | --- | --- | --- | --- | --- | --- | --- | --- | --- | --- |
| 3 | − | 8 | 99.3 ± 1.10 | 0.5 ± 0.76 | 61.5 | 88.9 | 97.9 | 0 | 5 | 0.6 ± 0.80 | 0.0* |
| 3 | + | 8 | 98.6 ± 3.99 | 0.7 ± 2.23 | 86.7 | 89.3 | 91.2 | 0 | 19 | 0.6 ± 1.93 | 4.7 |
| 5 | − | 8 | 99.3 ± 1.05 | 0.5 ± 0.86 | 63.5 | 87.3 | 95.9 | 0 | 5 | 1.1 ± 1.00 | 0.0* |
| 5 | + | 8 | 97.0 ± 6.13 | 1.4 ± 3.13 | 70.4 | 76.8 | 81.6 | 0 | 25 | 1.4 ± 3.75 | 9.9 |
| 8 | − | 8 | 100.0 ± 0.00 | 0.0 ± 0.00 | 100.0 | 100.0 | 100.0 | 0 | 0 | 0.0 ± 0.00 | 0.0 |
| 8 | + | 8 | 91.8 ± 8.26 | 4.0 ± 4.07 | 28.3 | 38.4 | 48.0 | 3 | 18 | 3.8 ± 4.95 | 20.8 |

*Analyzed with *Display “long” indels* option selected. With default parameters, the second insertion was reconstructed as *y*– *x* bp instead of *y*. For explanation see discussion on reconstruction of multiple indels in Materials and Methods and Fig. 5.
