# Supplementary material for: Decoding of Superimposed Traces Produced by Direct Sequencing of Heterozygous Indels
Source: PLoS Comput Biol. 2008 Jul 25;4(7):e1000113. doi: 10.1371/journal.pcbi.1000113 (PMC2429969; doi:10.1371/journal.pcbi.1000113)
Supplement: Table S4 — Decoded mixed human traces, and the number of errors, putative SNPs, and other ambiguous bases in the consensus reconstructions as revealed by BLASTN comparisons with sequences in the NCBI Trace Archive database. (0.21 MB DOC) [file pcbi.1000113.s004.doc]

**Table S4.** Decoded mixed human traces, and the number of errors, putative SNPs, and other ambiguous bases in the consensus reconstructions as revealed by BLASTN comparisons with sequences in the NCBI Trace Archive database.

| Trace name in NCBI Trace Archive | Chromosome | Length of indel,  bp | Length of reconstructed mixed trace, bp | Total errors | Errors  in indel | Errors  in last *k* sites | Errors confirmed as basecalling errors | SNPs | Ambiguities |
| --- | --- | --- | --- | --- | --- | --- | --- | --- | --- |
| 68483542 | 8 | 5 | 105 | 0 | 0 | 0 | 0 | 0 | 0 |
| 68959370 | 12 | 6 | 109 | 0 | 0 | 0 | 0 | 0 | 0 |
| 68965841 | 8 | 25 | 229 | 0 | 0 | 0 | 0 | 0 | 1 |
| 68967162 | 12 | 29 | 125 | 4 | 3 | 1 | 1 | 0 | 0 |
| 68969108 | 8 | 15 | 172 | 0 | 0 | 0 | 0 | 0 | 0 |
| 68973866 | 12 | 29 | 483 | 2 | 0 | 1 | 2 | 0 | 5 |
| 69521589 | 7 | 5 | 246 | 0 | 0 | 0 | 0 | 0 | 0 |
| 70210676 | 8 | 17 | 203 | 0 | 0 | 0 | 0 | 0 | 0 |
| 70427384 | 9 | 16 | 318 | 0 | 0 | 0 | 0 | 0 | 9 |
| 70756143 | 7 | 5 | 234 | 0 | 0 | 0 | 0 | 0 | 2 |
| 83516801 | 7 | 5 | 677 | 0 | 0 | 0 | 0 | 0 | 0 |
| 83822732 | 8 | 9 | 398 | 0 | 0 | 0 | 0 | 1 | 0 |
| 83878647 | 8 | 7 | 177 | 2 | 0 | 1 | 2 | 2 | 2 |
| 84259290 | 7 | 5 | 131 | 0 | 0 | 0 | 0 | 0 | 0 |
| 84368908 | 7 | 5 | 223 | 0 | 0 | 0 | 0 | 0 | 0 |
| 84382656 | 7 | 5 | 489 | 0 | 0 | 0 | 0 | 0 | 0 |
| 84446621 | 7 | 15 | 324 | 0 | 0 | 0 | 0 | 0 | 3 |
| 87117445 | 7 | 27 | 239 | 0 | 0 | 0 | 0 | 0 | 0 |
| 87153164 | 8 | 25 | 303 | 5 | 0 | 2 | 2 | 0 | 1 |
| 87192746 | 8 | 5 | 248 | 1 | 1 | 0 | 1 | 0 | 0 |
| 87636223 | 9 | 6 | 491 | 0 | 0 | 0 | 0 | 0 | 0 |
| 87756317 | 8 | 16 | 558 | 1 | 0 | 0 | 1 | 1 | 0 |
| 88585377 | 18 | 12 | 386 | 2 | 0 | 0 | 2 | 0 | 1 |
| 88586801 | 18 | 7 | 538 | 0 | 0 | 0 | 0 | 0 | 1 |
| 88622367 | 18 | 6 | 340 | 0 | 0 | 0 | 0 | 0 | 0 |
| 88665465 | 18 | 5 | 361 | 0 | 0 | 0 | 0 | 0 | 0 |
| 88744009 | 18 | 5 | 190 | 0 | 0 | 0 | 0 | 0 | 0 |
| 88852035 | 18 | 6 | 441 | 0 | 0 | 0 | 0 | 0 | 3 |
| 88977349 | 18 | 5 | 425 | 0 | 0 | 0 | 0 | 0 | 0 |
| 88991813 | 18 | 6 | 245 | 0 | 0 | 0 | 0 | 0 | 1 |
| 89058486 | 18 | 24 | 364 | 1 | 1 | 0 | 1 | 0 | 0 |
| 94146296 | 18 | 12 | 347 | 0 | 0 | 0 | 0 | 0 | 2 |
| 94150278 | 8 | 6 | 232 | 0 | 0 | 0 | 0 | 0 | 0 |
| 94574634 | 12 | 5 | 224 | 0 | 0 | 0 | 0 | 0 | 0 |
| 94585775 | 9 | 18 | 466 | 2 | 1 | 0 | 2 | 0 | 6 |
| 94800547 | 9 | 5 | 245 | 0 | 0 | 0 | 0 | 0 | 0 |
| 94801104 | 9 | 8 | 218 | 0 | 0 | 0 | 0 | 0 | 0 |
| 94801126 | 9 | 5 | 245 | 0 | 0 | 0 | 0 | 0 | 0 |
| 94888701 | 18 | 12 | 166 | 0 | 0 | 0 | 0 | 1 | 1 |
| 94889711 | 18 | 5 | 414 | 1 | 0 | 0 | 1 | 0 | 2 |
| 94955020 | 18 | 24 | 108 | 1 | 0 | 0 | 1 | 0 | 0 |
| 95176011 | 7 | 5 | 252 | 0 | 0 | 0 | 0 | 0 | 0 |
| C121P602FF2.T0 | 7 | 5 | 219 | 0 | 0 | 0 | 0 | 0 | 0 |
| C121P650RA8.T0 | 7 | 8 | 130 | 0 | 0 | 0 | 0 | 0 | 1 |
| C121P692RC7.T0 | 7 | 5 | 104 | 0 | 0 | 0 | 0 | 1 | 0 |
| C121P745RF12.T0 | 7 | 12 | 320 | 0 | 0 | 0 | 0 | 1 | 5 |
| C121P777RF10.T0 | 7 | 5 | 175 | 0 | 0 | 0 | 0 | 0 | 0 |
| C121P841FD1.T0 | 2 | 5 | 443 | 0 | 0 | 0 | 0 | 0 | 3 |
| C121P912RA4.T0 | 2 | 6 | 322 | 0 | 0 | 0 | 0 | 0 | 0 |
| C122P152FC3.T0 | 2 | 21 | 103 | 0 | 0 | 0 | 0 | 0 | 0 |
| C122P160RA3.T0 | 2 | 6 | 295 | 1 | 0 | 0 | 0 | 0 | 1 |
| C122P370RD10.T0 | 7 | 16 | 257 | 1 | 1 | 0 | 0 | 0 | 0 |
| C122P379RD10.T0 | 7 | 8 | 311 | 0 | 0 | 0 | 0 | 1 | 0 |
| C122P52RF10.T0 | 2 | 7 | 182 | 0 | 0 | 0 | 0 | 1 | 1 |
| C122P542FH3.T0 | 4 | 5 | 244 | 0 | 0 | 0 | 0 | 0 | 0 |
| C122P58RA11.T0 | 2 | 14 | 183 | 0 | 0 | 0 | 0 | 0 | 2 |
| C122P638FD1.T0 | 4 | 5 | 429 | 0 | 0 | 0 | 0 | 0 | 1 |
| C122P652FE8.T0 | 4 | 5 | 556 | 1 | 0 | 0 | 0 | 0 | 0 |
| C122P670FC6.T0 | 4 | 12 | 168 | 0 | 0 | 0 | 0 | 0 | 5 |
| C122P67FE12.T0 | 2 | 8 | 217 | 0 | 0 | 0 | 0 | 0 | 0 |
| C122P67RE12.T0 | 2 | 8 | 176 | 2 | 1 | 0 | 1 | 1 | 2 |
| C122P739RC3.T0 | 4 | 13 | 133 | 1 | 0 | 1 | 1 | 1 | 1 |
| C122P875RG9.T0 | 7 | 6 | 371 | 0 | 0 | 0 | 0 | 0 | 4 |
| C122P890FC5.T0 | 7 | 1 | 399 | 0 | 0 | 0 | 0 | 0 | 0 |
| C122P911FD8.T0 | 7 | 13 | 426 | 0 | 0 | 0 | 0 | 0 | 5 |
| C123P101FB9.T0 | 7 | 5 | 393 | 1 | 1 | 0 | 1 | 0 | 0 |
| C123P102FB8.T0 | 7 | 5 | 324 | 0 | 0 | 0 | 0 | 0 | 2 |
| C123P117FA6.T0 | 7 | 5 | 443 | 0 | 0 | 0 | 0 | 2 | 1 |
| C123P131RA10.T0 | 7 | 5 | 223 | 0 | 0 | 0 | 0 | 0 | 0 |
| C123P136RH12.T0 | 7 | 6 | 369 | 0 | 0 | 0 | 0 | 2 | 4 |
| C123P168RF3.T0 | 7 | 5 | 227 | 0 | 0 | 0 | 0 | 0 | 0 |
| C123P192RC6.T0 | 7 | 5 | 250 | 0 | 0 | 0 | 0 | 0 | 0 |
| C123P204RA8.T0 | 7 | 4 & 4 | 449 | 0 | 0 | 0 | 0 | 0 | 4 |
| C123P223RE8.T0 | 7 | 5 | 448 | 1 | 0 | 0 | 1 | 0 | 4 |
| C123P325RH2.T0 | 2 | 28 | 424 | 3 | 0 | 3 | 3 | 1 | 0 |
| C123P327RB7.T0 | 2 | 4 & 1 | 311 | 0 | 0 | 0 | 0 | 1 | 1 |
| C123P373RE4.T0 | 2 | 16 | 193 | 0 | 0 | 0 | 0 | 0 | 0 |
| C123P386RA9.T0 | 2 | 10 | 167 | 0 | 0 | 0 | 0 | 0 | 0 |
| C123P393RB1.T0 | 2 | 5 | 245 | 0 | 0 | 0 | 0 | 0 | 0 |
| C123P40RA7.T0 | 7 | 30 | 350 | 1 | 0 | 1 | 0 | 0 | 1 |
| C123P425FB10.T0 | 2 | 19 | 302 | 1 | 0 | 1 | 1 | 0 | 2 |
| C123P431RG2.T0 | 2 | 18 | 157 | 1 | 0 | 1 | 1 | 0 | 0 |
| C123P446RG8.T0 | 2 | 8 | 325 | 0 | 0 | 0 | 0 | 1 | 4 |
| C123P465FB1.T0 | 2 | 5 | 461 | 0 | 0 | 0 | 0 | 4 | 2 |
| C123P46FH12.T0 | 7 | 6 | 156 | 2 | 0 | 0 | 1 | 0 | 0 |
| C123P4RG8.T0 | 7 | 27 | 279 | 3 | 2 | 1 | 3 | 0 | 6 |
| C123P502FF3.T0 | 2 | 6 | 492 | 1 | 1 | 0 | 0 | 0 | 0 |
| C123P513FE1.T0 | 2 | 10 | 162 | 0 | 0 | 0 | 0 | 0 | 0 |
| C123P616FG4.T0 | 2 | 7 | 139 | 2 | 2 | 0 | 0 | 0 | 2 |
| C123P738FC3.T0 | 7 | 6 | 180 | 0 | 0 | 0 | 0 | 0 | 0 |
| C123P738FG11.T0 | 2 | 7 | 214 | 1 | 1 | 0 | 0 | 0 | 0 |
| C123P98FC2.T0 | 7 | 21 | 186 | 5 | 0 | 0 | 0 | 0 | 9 |
| C126P369RG8.T0 | 4 | 6 | 275 | 1 | 0 | 1 | 1 | 0 | 2 |
| C126P431RD11.T0 | 7 | 11 | 486 | 1 | 0 | 0 | 1 | 0 | 5 |
| C126P594FC2.T0 | 7 | 9 | 527 | 1 | 1 | 0 | 0 | 2 | 4 |
| C126P594RB2.T0 | 7 | 9 | 432 | 2 | 2 | 0 | 0 | 0 | 2 |
| C126P676FH6.T0 | 2 | 14 | 189 | 2 | 0 | 2 | 2 | 2 | 4 |
| C126P709RH6.T0 | 2 | 14 | 369 | 0 | 0 | 0 | 0 | 2 | 2 |
| C126P710FE4.T0 | 2 | 16 | 386 | 2 | 1 | 0 | 2 | 0 | 8 |
| C126P720RB12.T0 | 2 | 18 | 168 | 0 | 0 | 0 | 0 | 0 | 3 |
| C126P745FB7.T0 | 2 | 6 | 325 | 0 | 0 | 0 | 0 | 2 | 10 |
| C126P753RH5.T0 | 2 | 21 | 136 | 7 | 3 | 1 | 4 | 0 | 2 |
| C126P891FB5.T0 | 2 | 6 | 237 | 1 | 1 | 0 | 0 | 0 | 2 |
| C126P928RB10.T0 | 5 | 12 | 291 | 2 | 0 | 0 | 2 | 0 | 5 |
