# Supplementary material for: Decoding of Superimposed Traces Produced by Direct Sequencing of Heterozygous Indels
Source: PLoS Comput Biol. 2008 Jul 25;4(7):e1000113. doi: 10.1371/journal.pcbi.1000113 (PMC2429969; doi:10.1371/journal.pcbi.1000113)
Supplement: Table S5 — Completeness of reconstruction by ShiftDetector and by Indelligent, measured as the percentage of the input ambiguous sites decoded by each program. (0.08 MB DOC) [file pcbi.1000113.s005.doc]

**Table S5.** Completeness of reconstruction by ShiftDetector and by Indelligent, measured as the percentage of the input ambiguous sites decoded by each program.

| Trace name in  NCBI Trace  Archive | Length of indel, bp | Ambiguities  in input  (total length 100 bp) | Decoded by ShiftDetector,  % | Decoded by Indelligent,  % |
| --- | --- | --- | --- | --- |
| 68970745 | 4 | 78 | 71.8 | 100.0 |
| 69521589 | 5 | 74 | 67.6 | 100.0 |
| 69539046 | 4 | 82 | 74.4 | 100.0 |
| 70447604 | 4 | 44 | 68.2 | 100.0 |
| 70743495 | 4 | 76 | 63.2 | 96.1 |
| 84375674 | 4 | 66 | 74.2 | 100.0 |
| 84401940 | 4 | 79 | 82.3 | 100.0 |
| 87221536 | 4 | 75 | 62.7 | 100.0 |
| 87274655 | 4 | 69 | 66.7 | 100.0 |
| 87692005 | 4 | 69 | 69.6 | 100.0 |
| 87722845 | 4 | 71 | 67.6 | 100.0 |
| 88577478 | 4 | 78 | 73.1 | 97.4 |
| 88595276 | 4 | 64 | 70.3 | 100.0 |
| 88599518 | 4 | 70 | 68.6 | 98.6 |
| 88665465 | 5 | 70 | 77.1 | 100.0 |
| 88775230 | 4 | 69 | 71.0 | 98.6 |
| 88821100 | 4 | 72 | 76.4 | 94.4 |
| 88876199 | 4 | 71 | 80.3 | 100.0 |
| 88904793 | 4 | 78 | 68.0 | 98.7 |
| 88909765 | 4 | 67 | 73.1 | 94.0 |
| 94133643 | 4 | 71 | 63.4 | 100.0 |
| 94188458 | 4 | 78 | 75.6 | 97.4 |
| 94574634 | 5 | 73 | 65.8 | 100.0 |
| 94620223 | 4 | 70 | 74.3 | 100.0 |
| 94664654 | 4 | 74 | 75.7 | 100.0 |
| 94801126 | 5 | 72 | 81.9 | 100.0 |
| 94812293 | 4 | 67 | 59.7 | 100.0 |
| 94889711 | 5 | 62 | 83.9 | 100.0 |
| 95755235 | 4 | 71 | 74.7 | 97.2 |
| C121P316FC4.T0 | 4 | 69 | 75.4 | 100.0 |
| C121P602FF2.T0 | 5 | 69 | 85.5 | 100.0 |
| C121P612RC6.T0 | 4 | 75 | 62.7 | 98.7 |
| C122P542FH3.T0 | 5 | 74 | 73.0 | 100.0 |
| C122P542RF6.T0 | 4 | 74 | 81.1 | 94.6 |
| C122P564FD12.T0 | 4 | 72 | 75.0 | 97.2 |
| C122P588RB7.T0 | 4 | 74 | 78.4 | 100.0 |
| C122P652FE8.T0 | 5 | 74 | 71.6 | 98.7 |
| C122P665FH10.T0 | 4 | 65 | 78.5 | 100.0 |
| C123P146RG7.T0 | 4 | 50 | 56.0 | 96.0 |
| C123P168RF3.T0 | 5 | 75 | 70.7 | 100.0 |
| C123P204FB2.T0 | 4 | 70 | 72.9 | 92.9 |
| C123P327RB7.T0 | 5 | 68 | 64.7 | 98.5 |
| C123P427RA11.T0 | 4 | 69 | 81.2 | 100.0 |
| C123P458RB9.T0 | 4 | 78 | 61.5 | 100.0 |
| C123P54FC12.T0 | 5 | 71 | 78.9 | 100.0 |
| C123P633RA7.T0 | 4 | 76 | 69.7 | 100.0 |
| C123P780FF11.T0 | 4 | 77 | 76.6 | 100.0 |
| C126P323RB11.T0 | 5 | 75 | 77.3 | 93.3 |
| C126P337RH6.T0 | 4 | 72 | 69.4 | 100.0 |
| C126P419FA10.T0 | 4 | 76 | 75.0 | 100.0 |
| C126P467FD4.T0 | 5 | 73 | 71.2 | 100.0 |
| C126P709FB11.T0 | 5 | 69 | 71.0 | 100.0 |
| C126P900RB11.T0 | 5 | 72 | 79.2 | 100.0 |
| C126P953RH12.T0 | 4 | 73 | 78.1 | 100.0 |
